# Personality Traits, Perceived Stress, and Tinnitus-Related Distress in Patients With Chronic Tinnitus: Support for a Vulnerability-Stress Model

**DOI:** 10.3389/fpsyg.2019.03093

**Published:** 2020-01-24

**Authors:** Raphael Biehl, Benjamin Boecking, Petra Brueggemann, Romina Grosse, Birgit Mazurek

**Affiliations:** Tinnitus Center, Charité – Universitätsmedizin-Berlin, Berlin, Germany

**Keywords:** tinnitus, personality, vulnerability-stress, Tinnitus Questionnaire (TQ), perceived stress, FPI

## Abstract

**Background:**

Despite vulnerability-stress models underlying a variety of distress-related emotional syndromes, few studies have investigated interactions between personality factors and subjectively experienced stressors in accounting for tinnitus-related distress.

**Aim:**

The present study compared personality characteristics between patients with chronic tinnitus and the general population. Within the patient sample, it was further examined whether personality dimensions predicted tinnitus-related distress and, if so, whether differential aspects or levels of perceived stress mediated these effects.

**Method:**

Applying a cross-sectional design, 100 patients with chronic tinnitus completed the Freiburger Persönlichkeitsinventar (*FPI-R*) measuring personality, the Perceived Stress Questionnaire (*PSQ-20*) measuring perceived stress and the German version of the Tinnitus Questionnaire (*TQ)* measuring tinnitus-related distress. FPI-R scores were compared with normed values obtained from a representative German reference population. Mediation analyses were computed specifying FPI-R scores as independent, PSQ20 scores as mediating and the TQ-total score as dependent variables.

**Results:**

Patients with chronic tinnitus significantly differed from the general population across a variety of personality indices. Tinnitus-related distress was mediated by differential interactions between personality factors and perceived stress dimensions.

**Conclusion:**

In conceptualizing tinnitus-related distress, idiosyncratic assessments of vulnerability-stress interactions are crucial for devising effective psychological treatment strategies. Patients’ somatic complaints and worries appear to be partly informed by opposing tendencies reflecting emotional excitability vs. aggressive inhibition – suggesting emotion-focused treatment strategies as a promising new direction for alleviating distress.

## Introduction

Tinnitus is a symptom denoting the perception of acoustic sensations without an external sound stimulus. The prevalence in the general population ranges between 4 and 32%, and the levels of reported contemporaneous psychological distress vary considerably (Durai and Searchfield, 2016). Whilst some patients report depression and anxiety associated with the tinnitus percept (Schaaf et al., 2003; Durai and Searchfield, 2016), others report little or no tinnitus-related distress. Tinnitus can be acute or chronic with the latter being defined as a symptom duration of > 3 months (Deutsche Gesellschaft für Hals-Nasen-Ohren-Heilkunde Kopf- und Hals-Chirurgie, 2015). Depending on perceived tinnitus-related distress, tinnitus can also be divided into compensated and decompensated presentations with the latter involving high levels of tinnitus-related distress and associated symptoms of low mood and/or anxiety (Biesinger et al., 1998).

Whilst its causes are not always clearly identifiable and closely interlinked, chronic tinnitus has been associated with numerous risk factors (Haider et al., 2018; Trevis et al., 2018; Boecking et al., 2019) that have partly been interpreted within a vulnerability-stress framework. For example, emotional exhaustion and low emotional well-being were found to predict the risk of developing tinnitus (Hébert et al., 2012) with the former also being shown to predict higher sensitivity to sound following an acute stress task (Hasson et al., 2013). Moreover, several studies have shown that existing emotional distress predicted higher tinnitus-related distress (Bartels et al., 2009; Schaaf et al., 2014; Wallhäusser-Franke et al., 2015; Durai and Searchfield, 2016; Strumila et al., 2017; Sahlsten et al., 2018). On the other hand, high psychological resilience (i.e., an individual’s ability to adapt to adverse life conditions) was associated with higher emotional well-being that was – in turn – associated with lower tinnitus-related distress (Wallhäusser-Franke et al., 2014). In line with conceptualizations of other functional syndromes such as chronic pain (Flor, 1991; Linton, 2000; Wittchen and Hoyer, 2011), tinnitus-related distress might be conceptualized as a function of an interaction of pre-existing psychological vulnerability and life stressors that may include – but are not limited to – the tinnitus symptom.

Personality, i.e., the sum of an individual’s unique and stable aspects [i.e., personality traits] that describe, explain and predict one’s behavior (Asanger and Wenninger, 1999), is a well-established vulnerability factor for developing anxiety and depression following stressful experiences (e.g., Boyce et al., 1991; Kotov et al., 2010). Personality traits are psychological constructs that describe individual differences in perception, experience, emotion, cognition, and behavior on selected parameters. Personality factors could either render an individual vulnerable to developing tinnitus (e.g., Mucci et al., 2014), or facilitate the development and experience of psychological distress that, upon the perception of a tinnitus sound, extends toward the tinnitus percept (Peerenboom et al., 2015). Investigating personality factors bears high importance for understanding all psychological components of tinnitus-related distress and its maintenance, as personality may affect both exposure and reactivity to stressful events as well as differential choices of coping efforts and their differential effectiveness (Bolger and Zuckerman, 1995). Moreover, success rates of treatment approaches such as schema (Jacob and Arntz, 2013) or mentalization-based therapy (Vogt and Norman, 2018) increasingly refute the notion that personality-associated persistent emotional difficulties are stable. These treatments offer promising tools to address personality factors as modifiable treatment targets. Regarding tinnitus, some studies have investigated whether certain personality traits predict the presence or degree of tinnitus-related distress. For example, Weber et al. (2008) applied the Freiburg Personality Inventory (Freiburger Persönlichkeitsinventar, FPI-R, Fahrenberg et al., 2010) to a sample of 121 patients with chronic tinnitus and demonstrated significant differences in between patient groups with low and high tinnitus-related distress in the personality traits life satisfaction, excitability, aggressiveness, strain, somatic complaints, health concerns, and emotionality. Durai and Searchfield (2016) showed that tinnitus-related distress was associated with high neuroticism, low extraversion, high stress reaction, higher alienation, lower social closeness, lower well-being, lower self-control, lower psychological acceptance and presence of a type D personality, i.e., a tendency toward negative affectivity and social inhibition, and externalized locus of control. Moreover, several studies reported positive relations between tinnitus-related distress and a subset of “Big-Five” personality traits, namely low agreeableness, low extraversion and high neuroticism (Langguth et al., 2007; McCormack et al., 2014; Mucci et al., 2014; Dehkordi et al., 2015). Welch and Dawes (2008) stated alongside Durai et al. (2017) that compared to non-tinnitus control groups, tinnitus patients were more socially withdrawn, reactive to stress, and alienated as well as less self-controlled. Compared to an adult reference population, Chung et al. (2017) reported that tinnitus patients showed higher levels of harm avoidance and lower scores for novelty seeking, reward dependence, persistence, cooperativeness and self-transcendence. Overall, studies demonstrated mixed relations between tinnitus-related distress and a variety of personality factors. However, due to heterogeneous operationalizations of the investigated personality constructs, no consistent picture has of yet emerged.

The meaning of “stress” varies widely in the scientific field. It can describe external stimuli, the adaptive reaction to them or resulting physical or mental strain. Longitudinal studies that compare differential stress dimensions with regard to tinnitus or tinnitus-related distress do not yet exist (Boecking et al., 2019). However, several studies have investigated the interaction between personality traits and stressors as influencing psychological distress and somatic symptoms. Almeida (2005) used a diary method approach and reported that psychological resilience and sociodemographic factors predicted the likelihood of exposure, appraisal and reactivity to daily stressors. Personality traits can thus influence daily well-being through their interaction with stressors. Several other studies further suggest that subjects with high neuroticism are more likely to develop depressive symptoms upon exposure to daily hassles (Hutchinson and Williams, 2007; Vinkers et al., 2014; Hentrich et al., 2016) – to which help-seeking patients with tinnitus have also shown to be susceptible (Scott and Lindberg, 2000). Yang et al. (2013) reported that perfectionism – a trait known to be heightened in individuals with chronic tinnitus (Andersson et al., 2005) – predicted depression in interaction with achievement-related, but not interpersonal hassles. A few more studies showed that interactions between perfectionism, daily hassles or major life events had an influence on the occurrence and maintenance of depressive symptoms (Flett et al., 1997; Yang et al., 2013).

Overall personality traits interact with daily stressors in predicting psychological distress. Applying a psychological vulnerability-stress framework, the current study investigates how personality characteristics (as measured by the FPI-R) interact with perceived stress in explaining tinnitus-related distress in patients with chronic tinnitus.

### Hypotheses

We examined the following hypotheses:

(1) There are systematic differences in personality factors between patients with chronic tinnitus and the general population;
(2) There are systematic differences in personality factors between patients with decompensated and compensated chronic tinnitus; and
(3) Within patients with chronic tinnitus, the degree of tinnitus-related distress is a function of differential interactions between personality-factors and differing dimensions of perceived subjective stress.

## Materials and Methods

### Procedure

The current study included *N* = 100 patients with chronic tinnitus who had been referred to the Tinnitus Center at Charité – Universitätsmedizin-Berlin between 2011 and 2012 and who completed [1] the German version of the Tinnitus Questionnaire (TQ) measuring tinnitus-related distress, [2] the Freiburg Personality Inventory (FPI-R) measuring personality factors, and [3] the Perceived Stress Questionnaire – German modified version measuring perceived stress. The reference group for the FPI-R norms consists of 3740 non-institutionalized adult subjects who are representative of the German population (Fahrenberg et al., 2010). The study was carried out in accordance with the recommendations of the German S3 Guideline 017/064: Chronic Tinnitus (Deutsche Gesellschaft für Hals-Nasen-Ohren-Heilkunde Kopf- und Hals-Chirurgie, 2015). Data was collected as part of the clinic’s routine diagnostic procedures approved by the Ethics Committee of Charité Universitätsmedizin Berlin (Nr. EA 1/115/15). All participants gave written consent for the use of anonymized data for research purposes in accordance with the Declaration of Helsinki.

### Materials

#### Tinnitus Questionnaire – German Version (TQ; Goebel and Hiller, 1998)

The German version of the Tinnitus Questionnaire is a self-report questionnaire that measures the degree of tinnitus-related distress. The questionnaire consists of 52 items (“disagree” = 0, “partly agree” = 1, “agree” = 2), 40 items of which are included into the total score and two items being entered twice thus yielding a range between 0 and 84 points. The total score can be divided to reflect compensated (slight and moderate tinnitus-related distress, as defined by scores ranging from 0 to 46) and decompensated levels of tinnitus-related distress (severe and catastrophic, as defined by scores ranging from 47 to 84; Biesinger et al., 1998; Goebel and Hiller, 1998). The scale’s internal consistency is high (α = 0.95; Zeman et al., 2012).

#### Freiburg Personality Inventory (FPI-R, Freiburger Persönlichkeitsinventar; Fahrenberg et al., 2010)

The Freiburg Personality Inventory consists of 138 items (“not true” = 0, “true” = 1) across 12 personality dimensions that comprise 10 to 14 items each. The inventory has been validated across various languages and populations and the subscales’ internal consistencies are sufficient (α = 0.73–0.83; Fahrenberg et al., 2010). In the following, the dimensions will be explained in some detail to allow for a psychologically meaningful description of the patient sample. Descriptions have been translated and adapted from the FPI-R handbook (Fahrenberg et al., 2010, pp. 84–90).

*Life satisfaction* describes feelings of satisfaction, contentment with life, self-acceptance, and an optimistic vision of one’s own future. People with lower scores show discontent about past and present life conditions. They lack self-efficacy, tend to ruminate and are often fed up by their circumstances. They express gloomy and unhappy moods, depressiveness and a negative approach to life. People with higher scores are content about their life choices and conditions. They have high self-valuation and show optimism and a positive attitude toward life.

*Social orientation* describes social solidarity, i.e., one’s tendency to be generous, friendly, helping, and warm. Persons with low scores highlight individual responsibility regarding life conditions. They act selfish and with unsympathetic attitudes toward others. Persons with high scores feel a high social responsibility. They express helpfulness, react to worries of others, and are motivated to help, comfort and care. They also tend to feel guilty which motivates them to engage in helping others.

*Achievement orientation* describes a person’s ambition; wish to assert themselves, competition behavior, activism, and determination. Persons with low scores show low competitive behavior and very little ambition. Either because of principles against the competitive vision of life, or because professional and social achievements are not important life goals. People with high scores are achievement orientated and motivated. They are ambitious and solve problems fast and efficient. They also enjoy being in competition, in their profession and social life. Usually they show higher commitment to their profession than to leisure time activities.

*Inhibitedness* describes hesitant and shy behavior, which is characterized by withdrawal, inhibition, lack of self-confidence, and little development or verbalization capacities. Persons with low scores are easy-going, spontaneous and self-confident in social groups. Persons with high scores feel inhibited in social situations: they are afraid to enter rooms filled with other people, prefer to stay in the background, have difficulties to speak in front of others. They are easily embarrassed, often anxious and blush often. Interactions with strangers are difficult and hard for them. They have difficulties joining conversations or making friends.

*Excitability* describes impulsive behavior and lack of self-control – with slightly aggressive manifestations. Persons with a lower score are characterized by serenity. They are difficult to provoke or bother, stay calm and patient even in difficult and hectic situations with multiple disturbances. People with higher scores are easily irritated and worked up. They have difficulties to control their anger, show aggressive behavior in improvident statements. They react sensitive and rushed, even in unimportant situations.

*Aggressiveness* describes verbal or physical aggressive behavior. It describes mainly spontaneous reactive and dominating behaviors. Persons with lower scores show little aggression. They are either reserved, solitary, inhibited in expressing themselves or socially passive and can control their reactions. They do not use physical violence to enforce their needs or rights. Persons with higher scores show willingness to violent behavior. They can experience joy in rude jokes, showing up faults of others or hurting people. They defend themselves with fury and lack of control, perhaps even with physical violence, if they feel insulted or in their rights violated.

*Strain* describes a personal perception of subjective overload. This induces tension, stress, nervousness, and exhaustion. Persons with lower scores feel less stressed and overworked. They feel equal to their requirements and are able to fulfill their tasks. Persons with higher scores feel highly stressed: they have a lot of tasks, experience high requirements and time pressure.

*Somatic complaints* describe the subjective disturbance of one’s actual state of health. Persons with low scores rarely complain of physical symptoms. Persons with higher scores complain about sleeping disorders, headaches, meteoropathy, arrhythmia, hot flashes, cold extremities, an irritable stomach, a chest tightness, tics, and/or shivering.

*Health concerns* describe worries about one’s present and future state of health irrespective of the actual state of health. Persons with low scores show little worries about their own health. They are unconcerned, robust, and not over-protective. Persons with high scores describe a health orientated, worried behavior. They try to reduce risk of health-related harm, contagion, infection and accidents. They show hypochondriac tendencies, food and lifestyle control and often ask for medical or therapeutic advice.

*Frankness* describes open, unreserved and unconventional behaviors, which are characterized by straightforwardness. Persons with lower scores try to make good impressions with active impression management. Different motives can explain these behaviors: lack of self-criticism or self-idealization, reticence or conformity. People with higher scores are able to admit everyday mistakes or weaknesses: being late, procrastination, gloating, occasional lies, nasty thoughts, etc. They admit these deviations from the social norm without shame and do not see these norms as important or deviations as flagrant.

*Extraversion* describes one of the basic dimensions of most personality theories: it captures the difference between sociable, impulsive, active and socially present, dynamic and vivid persons, and reserved, uncommunicative, controlled, introvert ones. People with lower scores are withdrawn in social situations and prefer to be alone. They are calm and serious, uncommunicative, not enterprising and more likely self-controlled than impulsive. People with higher score are sociable and impulsive. They like to go out, varieties, entertainment, make friends fast, enjoy company of others and can be easy-going. They are active, communicative and eloquent in contact with others. They can be prankful, enterprising, energetic and ready to take command.

*Emotionality* describes the continuum of emotional stability to emotional lability and neuroticism. People with lower scores are satisfied with themselves and their life. They are serene, relaxed, and calm. They are little anxious or sensitive. They show mostly no health concerns, psychosomatic symptoms or inner conflicts. People with high scores show high numbers of problems and inner conflicts. They are excitable and irritable or feel tired, asthenic or indifferent. Their mood switches a lot, but they feel mainly depressed and anxious. They ruminate a lot and feel misunderstood by their peers and relatives. They are stressed, concerned about their health, nervous and psychosomatically accentuated.

#### Perceived Stress Questionnaire – German Modified Version (PSQ20; Fliege et al., 2005)

The Perceived Stress Questionnaire is a self-report questionnaire measuring perceived stress. The German modified version consists of 20 items with a four-point Likert-type scale (“almost never” = 1, “sometimes” = 2, “often” = 3, “usually” = 4; Fliege et al., 2005). Higher total scores indicate more severe perceived stress. Items are rated across four subscales: worries (worries, anxious concern for the future, and feelings of desperation and frustration), tension (disquietude, exhaustion and the lack of relaxation), joy (positive feelings of challenge, joy, energy, and security), and demands (perceived environmental demands, such as lack of time, pressure, and overload.). The resulting PSQ20 total und subscale scores are linearly transformed to scores ranging from 0 to 1. For the computation of the total score, the scale joy is inversed. The scale “demands” focuses on the subjective perception of external stressors, while the other three scales focus on internal stress reactions (Fliege et al., 2005). Originally designed in English, this instrument has been translated into French, Italian, German and Spanish, and validated in various populations (Kocalevent et al., 2007). The scale’s internal consistency is high (α = 0.90; Fliege et al., 2005).

### Participants

A total of *N* = 100 patients with chronic tinnitus (53% female) completed the TQ, FPI-R and PSQ20. On average, patients were 50 years old (*SD* = 12.38; range = 19–76). Seventy-three patients reported compensated tinnitus whilst 27 reported decompensated tinnitus. To interpret the reported FPI-R scores, scores were compared both with the reference population mean values published in the FPI-R – 8^*th*^ edition (*N* = 3740) (Fahrenberg et al., 2010) and between patients with compensated vs. decompensated tinnitus.

### Statistical Analysis

All analyses were conducted using IBM SPSS Statistics for Windows, version 24. Statistical significance was set at α = 0.05. For the comparisons of means, effect sizes (Cohen’s *d*) were also calculated. Effect sizes of Cohen’s d are defined as *d* (0.01) = very small, *d* (0.2) = small, *d* (0.5) = medium, *d* (0.8) = large, *d* (1.2) = very large, and *d* (2.0) = huge (Sawilowsky, 2009). *First*, we used descriptive statistics to explore sample descriptors. *Second*, we used the SPSS dummy matrix variable approach and independent samples *t*-tests to compare our sample means with the summarized data from the FPI-R population norms. *Third*, we used independent samples *t*-tests to compare decompensated and compensated patients. *Finally*, to explore interaction effects between personality traits (vulnerability) and perceived stress (stress) on tinnitus-related distress, mediation analyses were computed, specifying FPI-R dimensions as independent variables, PSQ20 dimensions as mediating variables and the TQ total score as dependent variable. Here, the PROCESS macro (Hayes, 2018) was used to compute a series of path coefficients: the effect of the independent variable X on the dependent variable Y (total effect, *c*); the effect of X on the mediator M (path *a*); the effect of M on Y (path *b*); the indirect effect (*ab*); and the total effect adjusted for *ab* (direct effect, *c*′). Whenever the effect of X on Y decreases to zero once M is included in the model, “complete mediation” is said to have occurred (James and Brett, 1984). In this case, there is strong evidence that the investigated mediator dominantly accounts for almost all variance in the outcome variable. “Partial mediation” is said to have occurred, if the effect of X on Y decreases significantly, but not necessarily to zero (Judd and Kenny, 1981). In the results section, indirect effects will be reported graphically – for an overview of estimates, see Appendix A.

## Results

### Descriptive Statistics

Table 1 shows sociodemographic factors and means for the TQ (German version), FPI-R, and PSQ20.

**TABLE 1:** Sample description.

	*N*	*M*	*SD*	Min	Max
***Gender***					
Male	47				
Female	53				
***Age***	100	50.00	12.38	19	76
***TQ_Total score***	100	33.71	16.80	0	73
***FPI-R***					
Life satisfaction	100	7.01	3.28	0	12
Social orientation	100	7.52	2.40	1	12
Achievement orientation	100	7.15	2.68	0	12
Inhibitedness	100	5.57	3.14	0	12
Excitability	100	6.81	3.10	0	12
Aggressiveness	100	3.42	2.42	0	11
Strain	100	7.51	3.80	0	12
Somatic complaints	100	4.31	2.38	0	10
Health concerns	100	5.50	2.79	0	12
Frankness	100	5.81	2.86	1	12
Extraversion	100	6.47	3.54	0	14
Emotionality	100	7.36	3.61	0	14
***PSQ20***					
Total	99	0.44	0.22	0.01	0.92
Worries	99	0.39	0.25	0.00	1.00
Tension	99	0.52	0.26	0.00	1.00
Joy*	99	0.53	0.26	0.00	1.00
Demands	99	0.50	0.28	0.00	1.00

M, mean; Min, minimum; Max, maximum; SD, standard deviation; TQ, Tinnitus Questionnaire (German version); PSQ20, Perceived Stress Questionnaire. *Higher values indicate more joy; for the total score, the coding is reversed.

### Comparison of Means

**FIGURE 1:**
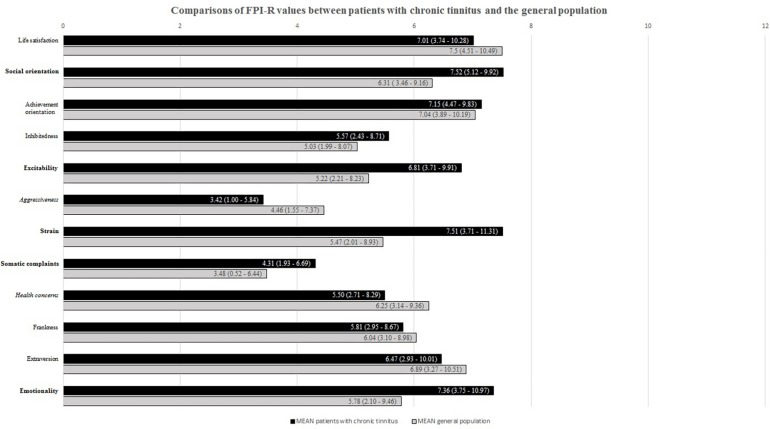
FPI-R values for patients with chronic tinnitus and the general population. Compared to the general population, bold labels indicate significantly higher; italicized labels significantly lower scores for patients with chronic tinnitus.

First, we compared FPI-R mean values of tinnitus patients to those of the general population. For the tinnitus patients, results showed significantly elevated values in [+] social orientation (*p* = 0.000, *d* = 0.426), excitability (*p* = 0.000, *d* = 0.528), strain (*p* = 0.000, *d* = −0.588), somatic complaints (*p* = 0.000, *d* = 0.282), emotionality (*p* = 0.000, *d* = 0.430), and significantly lower values in [−] aggressiveness (*p* = 0.000, *d* = −0.359) and health concerns (*p* = 0.000, *d* = 0.426) (see Figure 1). Differences in social orientation, aggressiveness, somatic complaints, health concerns and emotionality yielded small effect sizes; differences in excitability and strain medium effect sizes. We then explored Pearson correlations between the personality dimensions that distinguished tinnitus patients from the general population in our sample. Here, coefficients suggested an affectively centered cluster comprising strong correlations between emotionality and excitability, strain and somatic complaints (see Table 2).

**TABLE 2:** Intercorrelations of factors that distinguish patients with chronic tinnitus from the general population.

	Aggressiveness	Strain	Somatic complaints	Emotionality
Aggressiveness				0.275**
Strain			0.479**	0.726**
Somatic complaints				0.582**
Excitability	0.256*	0.495**	0.338**	0.616**

Only significant coefficients are reported. *p < 0.05, **p < 0.01 (two-tailed). Italicized values denote small (below ± 0.29), underlined values medium (± 0.30 and ± 0.49), and bold values strong correlations (± 0.50 and ± 1).

Third, we compared FPI-R values between decompensated and compensated tinnitus patients. Results showed significantly higher values in [+] excitability, strain, somatic complaints, and emotionality alongside significantly lower values in [−] life satisfaction (Table 3). Medium effect sizes emerged for life satisfaction, excitability and strain; large effect sizes for emotionality and somatic complaints.

**TABLE 3:** Comparisons of FPI-R values between patients with decompensated and compensated chronic tinnitus.

FPI-R scale	*Decompensated* tinnitus patients *n* = 27	*Compensated* tinnitus patients *n* = 73	*p*	*d*
**Life satisfaction**	**5,67 ± 3,15**	**7,51 ± 3,20**	**0.012**	**−0,577**
Social orientation	7,85 ± 2,41	7,40 ± 2,40	0.408	
Achievement orient.	7,22 ± 2,91	7,12 ± 2,60	0.869	
Inhibitedness	5,41 ± 3,24	5,63 ± 3,13	0.758	
**Excitability**	**7,89 ± 2,67**	**6,41 ± 3,18**	**0.034**	**0,485**
Aggressiveness	3,56 ± 2,58	3,37 ± 2,37	0.729	
**Strain**	**9,26 ± 2,40**	**6,86 ± 4,03**	**0.005**	**0,654**
**Somatic complaints**	**6,11 ± 1,74**	**3,64 ± 2,24**	**0.000**	**1,166**
Health concerns	5,26 ± 2,10	5,59 ± 3,02	0.603	
Frankness	5,56 ± 2,98	5,90 ± 3,02	0.617	
Extraversion	6,67 ± 3,93	6,40 ± 3,41	0.737	
**Emotionality**	**9,41 ± 2,58**	**6,60 ± 3,66**	**0.000**	**0,825**

*Bold values denote significant differences between the groups, *p* < 0.05.*

### Mediation Analyses

**FIGURE 2:**
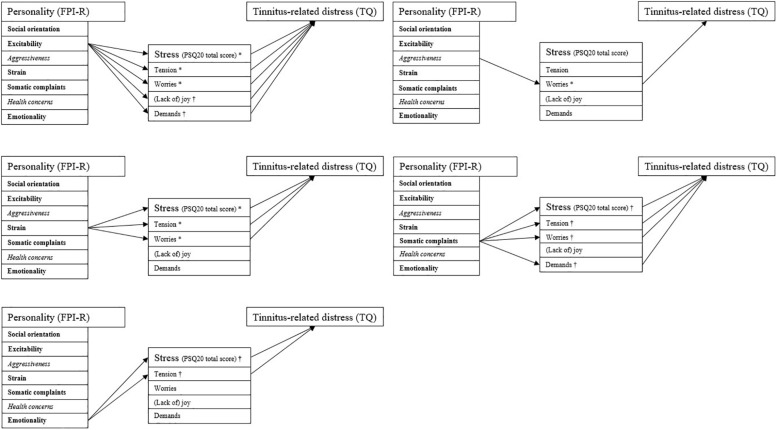
Indirect effects of the mediation analyses. The PSQ20 subscale “joy” was inverted for reasons of consistent presentation (i.e., higher scores indicating higher perceived stress). Arrows indicate significant indirect effects (*p* < 0.05). All path coefficients are positive. Compared to the general population, bold labels indicate significantly higher; italicized labels significantly lower scores for patients with chronic tinnitus. ^∗^Complete mediation (total effect reduced to non-significance upon inclusion of *ab*); ^†^ partial mediation (total effect not reduced to non-significance upon inclusion of *ab*).

Exploring possible interactions of vulnerability (personality dimensions) and stress (perceived stress) factors in predicting tinnitus-related distress, we computed sets of mediation analyses specifying those personality factors as independent variables that were found to significantly differ for tinnitus patients compared to the general population (cf. Figure 1). As mediators, we specified the total and subscale scores of the PSQ20 questionnaire with the dependent variable being specified as tinnitus-related distress as measured by the TQ total score. Figure 2 shows the significant effects of the explorative mediation analyses.

Overall, the following indirect effects accounted for the relationship between personality factors and tinnitus-related distress:

(1) For personality traits that were significantly more pronounced in patients with chronic tinnitus compared to the general population:

- Higher excitability interacting with (a) higher perceived tension, (b) higher worries, (c) less joy, and (d) higher demands;
- Higher strain interacting with (a) higher perceived tension and (b) higher worries;
- Higher somatic complaints interacting with (a) higher perceived tension, (b) higher worries, and (c) higher demands; and
- Higher emotionality interacting with (a) higher perceived tension.

(2) For personality traits that were significantly less pronounced in patients with chronic tinnitus compared to the general population:

- Higher aggressiveness interacting with (b) higher worries.

Social orientation and health concerns did not interact with perceived stress in predicting tinnitus-related distress. Appendix A reports the detailed results of the mediation analyses (a three-step logistic regression analysis) outlining coefficients “*a*” (effects of the independent variables on the mediators), “*b*” (effect of the mediators on the dependent variable), “*c*” (total effect of the independent variable on the dependent variable), “*c*′” (direct effect; i.e., the total effect adjusted for the indirect effect) and the indirect effect “*ab”* that is tested for significance using a *bootstrapping* approach yielding 95% confidence intervals.

## Discussion

The present study investigated interrelations between personality factors as measured by the FPI-R, perceived stress (PSQ20) and tinnitus-related distress (TQ-German version) in a sample of 100 patients with chronic tinnitus.

### Comparisons Between Tinnitus Patients and Between Tinnitus Patients and the General Population

Hypothesis 1: There are systematic differences in personality factors between patients with chronic tinnitus and the general population.

Results of this study indicate differences in personality traits between patients with chronic tinnitus and the general population as measured using the FPI-R. Compared to the general population, patients rated themselves as [1] experiencing higher social responsibility and reacting more readily to the worries of others (+ social orientation), [2] being more easily irritated, worked up, sensitive and rushed – with slight aggressive manifestations (+ excitability), [3] having a substantively higher personal perception of subjective overload; including habitual stress, nervousness and exhaustion (+ strain), [4] complaining more about somatic symptoms (+ somatic complaints), [5] being more excitable and irritable *or* tired, asthenic or indifferent and feeling not understood by their peers and relatives (+ emotionality), [6] being more inhibited in expressing themselves and socially passive (− aggressiveness), and, [7] being *less* worried about their personal state of health – possibly underlying fewer health-orientated behaviors (− health concerns).

The results are partly in keeping with previous studies researching relations between tinnitus-related distress and personality factors: in particular, patients’ higher emotionality and excitability scores support previous findings reporting higher scores of neuroticism and type D personality characteristics (e.g., Langguth et al., 2007; McCormack et al., 2014; Mucci et al., 2014; Durai and Searchfield, 2016) thereby supporting the importance of these constructs as risk factors for tinnitus-related distress.

Hypothesis 2: There are systematic differences in personality factors between patients with decompensated and compensated chronic tinnitus.

In keeping with results from the comparisons of the overall sample with the general population, patients with decompensated (vs. compensated) tinnitus yielded higher expressions of excitability, strain, somatic complaints and emotionality and lower expressions of life satisfaction. By contrast, we did not find differences in aggressiveness and health concerns between the two subpopulations. These results partly reflect previous findings from Weber et al. (2008) who compared tinnitus patients across severity grades I–IV of the Tinnitus Questionnaire and reported that, compared to grade I patients, grade IV patients had lower life satisfaction and higher excitability, aggressiveness, somatic complaints, and emotionality ratings whilst health concerns were found to differ between grade I and III patients only.

Overall, patients with chronic tinnitus show predispositions toward interpreting and responding to stimuli in a manner characterized by easy irritation, high levels of subjective overload, inner conflict, and higher ruminative tendencies whilst being more inhibited in expressing their emotional needs alongside a guilt-associated tendency to orientate themselves toward others’ needs. Interestingly, patients also report a lower degree of health concerns that might interact with higher excitability and higher social orientation in reflecting a coping style potentially aiming to regulate unexpressed emotion such as inhibited aggressivity (e.g., not using hearing protection). Relative to comparisons between tinnitus patients and the general population, the subsample of patients with *decompensated* tinnitus showed a somewhat similar, yet more pronounced profile across the excitability, strain, somatic complaints, and emotionality dimensions. Whilst the distinction between patients with compensated vs. decompensated tinnitus is clinically common (e.g., Stobik et al., 2005; Graul et al., 2008; Heinecke et al., 2008), results of the present study challenge the helpfulness of this dichotomization. Rather, personality traits appear to inform state cognitive-affective reactions to stimuli along a *continuum* of vulnerability-stress interactions with decompensation indicating a more pronounced expression of underlying, yet comparable, processes.

### Vulnerability-Stress Interactions

Hypothesis 3: Within patients with chronic tinnitus, the degree of tinnitus-related distress is a function of differential interactions between personality-factors and differing dimensions of perceived subjective stress.

While personality constructs are understood as comparably stable traits of a person, perceived stress – as measured in the present study – can be understood as reflecting negative state stress-related perceptions. The results of the mediation analyses may thus represent vulnerability-stress interactions that contribute to tinnitus-related distress yet do not, however, allow for assuming causality. Results indicated that tinnitus-related distress was predicted by vicious cycles between dispositional patterns of feeling easily irritated, strained, and emotional which interact with state experiences of high perceived stress, in particular emotional tension and worries, in response to a variety of stimuli. Thus, tinnitus-related distress appears to be one possible expression of distress within a broader experience of dispositional stimulus-processing and behavioral patterns associated with psychological distress (or the inhibition thereof) and mild risk-taking behaviors.

The relationship between aggressiveness and tinnitus-related distress was found to be mediated by worries against the background of an overall inhibited expression of aggressiveness relative to the general population. Placing this finding in context, patients’ high levels of concerns for others, inhibited expression of aggression, and *lower* levels of health anxiety and –related safety behaviors suggest that patients’ high levels of worries may be less indicative of illness concerns (of which they express many), but may instead reflect *internal coping attempts to regulate aggressive tendencies*. On the extreme end of this spectrum, vicious cycles between high degrees of (suppressed) aggressiveness, high impulsivity, and high social orientation would be reflected in a clinical presentation of a self-sacrificing patient reporting high levels of tinnitus-related distress and worries that he/she might be attributing to the tinnitus sound, yet which may instead reflect unexpressed aggressive tendencies stemming from a felt need for behaving socially desirable in the face of possibly challenging interpersonal circumstances.

Interestingly, only the relationships between excitability and somatic complaints on tinnitus-related distress were mediated by demands (i.e., the *internal perception of external* stressors). By contrast, most other effects were mediated by patients’ experiences of their *internal stress reactions* – notably *emotional tension* and *worries*. We believe that *emotional tension* reflects an affective state that patients with chronic tinnitus attempt to regulate through cognitive avoidance expressing itself in high levels of worry (Borkovec et al., 2004). Clinically, this lends support to the hypothesis that the *inner experience* of distress associated with patients’ broader life situations may form a primary target for case conceptualization and intervention in patients with chronic tinnitus. By contrast, patients’ frequently reported emphases of somatic symptoms or external stressors should be understood as emotion regulation attempts that are possibly informed by patients’ needs for interpersonal support and validation upon struggling with guilt or distress-informed ways of interpreting their internal and external worlds.

Overall, the observed interaction patterns highlight the importance of considering personality traits in interaction with state experiences when trying to explain and treat tinnitus-related distress on a general or individual level. Whilst several studies have demonstrated effects of cognitive-behavioral therapy (CBT) interventions that included “stress management” techniques (Cima et al., 2014), the individual conceptualization of perceived “stress” in the context of dispositional personality traits appears crucial in understanding and meeting the needs of patients with chronic tinnitus. These idiosyncratic conceptualizations ought to consider *individual interactions of early experiences and personality traits, and their situational activation and expression across different stimulus-processing contexts that may include, but are not limited to the tinnitus sound* thus allowing for individualized case conceptualizations and derived treatment strategies.

Psychological interventions that aim to encourage and facilitate emotional expression and -regulation may successfully reduce “emotional tension” thus providing a protective shield in the face of perceived stressors – even in the face of more stable personality traits indicating high vulnerability. Crucially, psychological interventions should focus on the *symptom function*, *affective states* and *difficulties in emotion regulation* that are likely to underlie observed (and commonly reported) worries about the tinnitus sound – and not necessarily attempt to address the worries’ content “at face value” only. If indicated, treatment approaches should further address personality factors that predispose individuals to reacting toward a broad range of stimuli with high levels of perceived distress. There is now good evidence that personality factors continue to change in adulthood (Roberts and Mroczek, 2008) and psychological treatment approaches for personality problems have shown considerable effects (Cristea et al., 2017). Here, treatment frameworks that are based on third-generation behavior therapy models such as Compassion-Focused Therapy (CFT; Gilbert, 2010) or Schema Therapy (Young et al., 2003) provide useful bases for addressing more engrained stimulus-processing patterns and have been shown to meaningfully improve depression and anxiety-related difficulties (Leaviss and Uttley, 2015; Taylor et al., 2017). Although these approaches have not yet been trialed in patients with chronic tinnitus, preliminary evidence suggests their potential conceptual relevance in patients with somatization disorder (e.g., Davoodi et al., 2018); however, respective research strands are in their infancy.

### Limitations

The current study has several limitations: in comparing patients’ ratings with the general population, it cannot be ruled out, that a proportion of the FPI-R reference population might have also suffered from tinnitus symptomatology. However, the representative sample was normed against criteria including “state of health,” “chronic illness,” “hospital admissions,” “doctor appointments,” and “psychological therapy,” rendering an above-chance proportion of chronic tinnitus patients unlikely to have been included. Moreover, whilst clinically common, the subdivision of patients into subgroups with compensated vs. decompensated tinnitus yields several disadvantages. These include, for example, the loss of statistical information and potential miscategorizations of patients close to the cut-off point as different rather than similar (Purgato and Barbui, 2013). The mediation analyses, by contrast, conceptualize tinnitus-related distress as a continuous variable. Owing to the cross-sectional design of the study, however, temporal lags between the formation of personality traits and their interaction with currently perceived stress cannot be established. Similarly, mediation analyses neither imply nor allow for assumptions of causality. Intercorrelations between habitual processing styles and state perceived stress variables are likely confounded; however, provide two different-yet-related targets for reducing tinnitus-related distress within psychological treatment frameworks.

## Conclusion

Individual personality traits and their differential interactions with subjective experiences of internal or external stimulus-processing contexts provide valuable targets for assessments, case-conceptualizations, and treatments of patients with chronic tinnitus. Whilst the literature on personality factors and tinnitus-related distress is mixed, theorization and empirical investigation of vulnerability-stress models offers a more nuanced and ultimately more meaningful way of modeling and predicting tinnitus-related distress within a broader psychological conceptualization framework. Moreover, psychological trait x state models offer helpful ways of identifying and clustering patient-subpopulations that may benefit from respectively matched treatment protocols. Future studies ought to conceptualize tinnitus-related distress and psychological trait and state variables as continuous, interacting factors in order to predict, prevent or treat maladaptive exacerbations of psychological distress pathways.

## Data Availability Statement

The datasets generated for this study are available on request to the corresponding author.

## Ethics Statement

The studies involving human participants were reviewed and approved by the Charité Universitätsmedizin Berlin EA 1/115/15. The patients/participants provided their written informed consent to participate in this study.

## Author Contributions

RB: literature review, data analysis, data interpretation, and co-wrote article (Introduction, Materials and Methods, and Results). BB: literature review, devised data analysis strategy, data analysis, data interpretation, wrote substantive section of article (Abstract, Introduction, Materials and Methods, and Discussion), and addressed reviewer comments. PB: idea for study conceptualization/design, first data analysis, and commented on previous draft of the manuscript. RG: responsible for data collection. BM: idea for study conceptualization/design, supervision of publication, and head of department.

## Conflict of Interest

The authors declare that the research was conducted in the absence of any commercial or financial relationships that could be construed as a potential conflict of interest.

## APPENDIX

**TABLE A1:** Path coefficients, confidence intervals and indirect effects for mediation analyses with FPI-R [independent variables], PSQ20 [mediators] and TQ total score indices [dependent variable].

FPI-E	PSQ20	Path *a*	*LLCI*	**ULCI**	Path *b*	*LLCI*	*ULCI*	*ab*	*LLCI*	*ULCI*	Path *c*	*LLCI*	*ULCI*	Path *c’*	*LLCI*	*ULCI*
Excitability	total	0.0381	0.0264	0.0497	36.7785	21.0639	52.4931	1.4001	0.7353	2.1866	2.0471	1.0468	3.0473	0.6470	−0.4408	1.7349
	worries	0.0414	0.0276	0.0551	28.8191	15.3195	42.3186	1.1919	0.6227	1.9099	2.0471	1.0468	3.0473	0.8552	−0.2235	1.9339
	tension	0.0419	0.0274	0.0565	28.7590	16.0916	41.4264	1.2057	0.6152	1.9383	2.0471	1.0468	3.0473	0.8414	−0.2153	1.8982
	joy	−0.0399	−0.0543	−0.0254	−19.8637	−33.2608	−6.4666	0.7916	0.1728	1.5300	2.0471	1.0468	3.0473	1.255	0.1543	2.3566
	demands	0.0359	0.0193	0.0525	16.6896	4.9618	28.4114	0.5997	0.1297	1.1395	2.0471	1.0468	3.0473	1.4474	0.3933	2.5015
Aggressiveness	worries	0.0221	0.0020	0.0422	34.2950	22.3140	46.2761	0.7576	0.0367	1.6186	0.7878	−0.5889	2.1645	0.0302	−1.1960	1.2564
Strain	total	0.0456	0.0388	0.0523	37.3904	15.1495	59.6312	1.7032	0.6465	2.8026	2.0217	1.2364	2.8070	0.3185	−0.9405	1.5774
	worries	0.0435	0.0337	0.0534	24.7134	9.3000	40.1269	1.0760	0.3779	1.8164	2.0217	1.2364	2.8070	0.9457	−0.0614	1.9528
	tension	0.0521	0.0432	−0.0610	26.8539	9.7738	43.9340	1.3981	0.5777	2.3826	2.0217	1.2364	2.8070	0.6230	−0.5421	1.7881
Somatic	total	0.0474	0.0317	0.0631	23.8626	10.0870	37.6382	1.1313	0.4422	1.8991	4.3041	3.1714	5.4367	3.1727	1.9154	4.4301
complaints	worries	0.0537	0.0355	0.0720	18.2776	6.2707	30.2844	0.9821	0.3330	1.7301	4.3041	3.1714	5.4367	3.3220	2.0570	4.5870
	tension	0.0575	0.0386	0.0764	18.3932	6.8327	29.9537	1.0581	0.4651	1.7433	4.3041	3.1714	5.4367	3.2460	1.9745	4.5175
	demands	0.0412	0.0188	0.0636	11.9519	1.9955	21.9082	0.4921	0.0805	1.0327	4.3041	3.1714	5.4367	3.8120	2.6321	4.9919
Emotionality	total	0.0456	0.0380	0.0533	23.0133	2.9236	43.1031	1.0500	0.0164	1.9647	2.5071	1.7300	3.2842	1.4571	0.2657	2.6484
	tension	0.0504	0.0402	0.0606	18.0812	3.1095	33.0529	0.9109	0.1592	1.6423	2.5071	1.7300	3.2842	1.5962	0.5264	2.6661

*All analyses were computed using the PROCESS macro (Hayes, 2018); resampling procedures (bootstrapping) comprised 10000 replicates. *LLCI* = Lower level confidence interval (95%), *ULCI* = Upper level confidence interval (95%). Path *a* denotes the effect of the independent variable on the mediator; path *b* the effect of the mediator on the dependent variable; *ab* denotes the product term, i.e., indirect effect. Path *c* denotes the *total* effect of the independent variable on the dependent variable; path *c*′ the *direct* effect; i.e., the total effect adjusted for *ab*.*
